# How has the Olympic legacy transformed the heart of East London? Understanding socio-economic exclusions and disproportionate COVID-19 impact on minoritised communities through a rights-based perspective

**DOI:** 10.3389/fspor.2023.1170466

**Published:** 2023-07-20

**Authors:** Farjana Islam

**Affiliations:** The Urban Institute, School Energy Geoscience Infrastructure and Society Heriot-Watt University, Edinburgh, United Kingdom

**Keywords:** COVID, ethnic minority, East London, legacy 2012, Olympics, inequality, British-Bangladeshi, Black African Caribbean

## Abstract

This research paper explores the experience of British-Bangladeshi and Black African Caribbean communities living in the areas surrounding London's Olympic Park, in terms of how they are appropriating the legacy-led socio-spatial changes, applying Lefebvre's right to the city perspective. Highlighting the top-down legacy-led regeneration process, the empirical evidence suggests that the games-led regeneration is contributing to an unjust trade-off between pre-existing minoritised ethnic residents and wealthier gentrifiers, ignoring the real needs of the socially and economically disadvantaged ethnic minority communities in East London. The findings provide a further understanding of factors such as housing and health-related inequalities and sub-standard living conditions, which may have contributed to the disproportionate impact of COVID-19 on the Bangladeshi and African Caribbean people living in East London boroughs. Given the scale of the pandemic, the paper argues that a greater understanding of the socio-structural problems and barriers arising out of poverty and deprivation is needed in order to formulate appropriate policy interventions to reduce disproportionate social, economic and health-related impacts on some minoritised communities, which could be achieved through residents' active participation and appropriation at different stages of the legacy-led regeneration process.

## 1. Introduction

Being a global city, London has always been transformed and retrofitted as a symbol of imperialism and global power underpinned by elitist interests. In the aftermath of British colonialism, London retained strong links with Commonwealth member states, and therefore from the 1950, many people from new Commonwealth nations migrated to the UK in response to Britain's calls to contribute to its workforce and rebuild the UK's postwar infrastructure and economy. Many of these migrants settled in the east end of London because of cheap accommodation, and job opportunities in the docks and the sweated trades and gradually the docklands and the surrounding east end became the most culturally and ethnically diverse part of the city (1, 2). With the events of deindustrialisation from the 1980s, these Commonwealth immigrants became unemployed or shifted to marginally paid jobs (3). The younger generations with a heritage from new Commonwealth countries, who are generally more educated and skilled than their older generations, are also trapped in poverty and structural racism by pursuing low-paid and unsecured jobs (4–6). Therefore, the longstanding Black and Asian ethnic minority communities have become the *de facto* post-industrial “working class” in London (3, 7) and remained excluded and segregated in the deprived areas along the River Lea in East London. In this way, the Olympic host boroughs are known to contain the poorest ethnic minority population in London and display a spatial concentration of sub-proletarian poverty and inequality (8).

The longstanding minoritised communities are usually concentrated in deprived parts of the inner-city and have experienced longstanding socio-economic exclusions in terms of health and housing-related inequalities compared to the mainstream White-British population. In terms of health inequality at the national level, Chouhan and Nazroo (9) summarised that ethnic minority groups self-reported their poor health more frequently than the White groups according to the 1999 Health Survey analysis. In relation to housing, the Government Ethnicity Facts and Figures website^1^ suggested that 24% of Bangladeshi households and 23% of Black African and Caribbean households were overcrowded compared to 2% White-British households. The Government Ethnicity Facts and Figures also show that a higher percentage of Bangladeshi and Black African Caribbean households were likely to have damp problems than White-British households. Such poorer health conditions and housing inequalities, together with occupations which have higher exposures to virus transmission, have exacerbated the pre-existing health inequalities and heightened the disproportionate COVID-19 impacts brought about by different coronavirus variants throughout the pandemic. The Olympic host borough Newham, which contains the stadium and other games infrastructure, was one of the worst affected boroughs in London, followed by two other host boroughs – Hackney and Tower Hamlets – in terms of the higher number of confirmed cases during the first wave. One of the reasons for the high mortality rates in London and particularly in the Olympic host boroughs could be the ethnic composition of the population because and 42% of the population who lives in four host boroughs (i.e., Hackney, Newham, Tower Hamlets and Waltham Forest) are from ethnic minority backgrounds (10).

**Figure 1:**
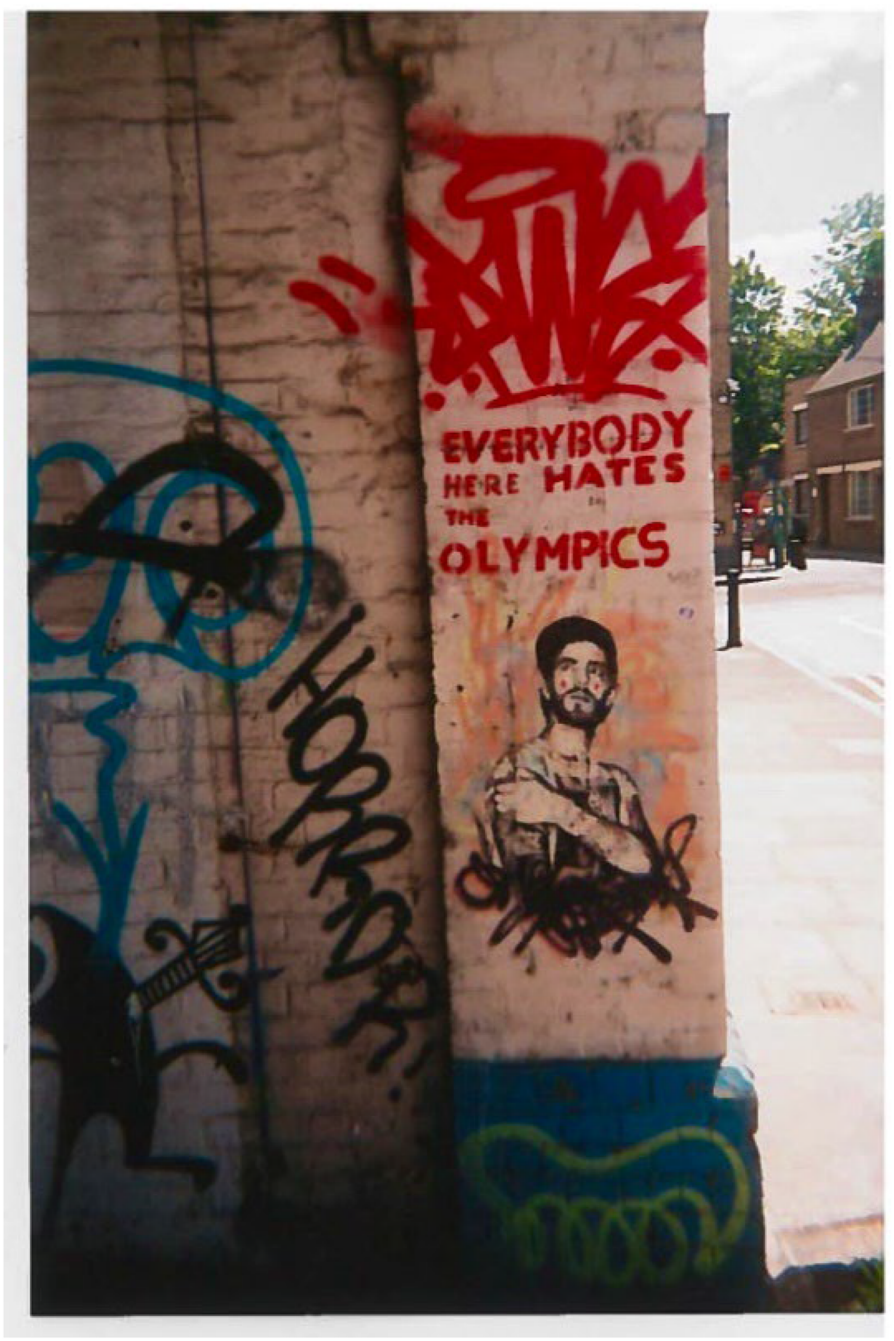
Graffiti in the peanut factory in hackney wick from 2012. *“Graffiti in the peanut factory from 2012. The Olympics look great on television, but for the residents, they are painful. A gigantic construction site on our doorstep with dust, dirt and noise. Lots of hype & promise, especially for local businesses. Then a two-week event which we are entirely shut off from, helicopters circling around US day and night followed by a complete shutdown of the area again for several years. The graffiti had a little addition which is now not visible anymore: Everybody here hates the Olympics (except for the 100 metres). The yellow on blue at the bottom is done by one of Hackney Wick's most prominent graffiti artists, Sweet Toof”* (Photo-elicitation interview with Mr. Becker, an artist in Hackney Wick).

At the time of bidding to host the Summer Olympic Games of 2012, the eastern boroughs to the north of the river (Hackney, Tower Hamlet, Waltham Forest, Newham) were listed as the most deprived boroughs in London (English Indices of Deprivation, 2010). To redress the spatial imbalances across the capital, the London Plan 2004 set out a range of policies with “an overall priority to East London” (11). When London won the Olympic bid, The Olympic Games were seen as a catalyst to regenerate the area surrounding the Olympic Park with the rhetoric of “developing the heart of East London” to benefit the historically deprived ethnic minority communities in adjacent host boroughs, such as Hackney, Newham, and Tower Hamlets. The plan was to create jobs and housing with a view to minimising the deprivation gap between the host boroughs and the rest of London. However, the Index of Multiple Deprivation (IMD2015) published after the Games suggested that deprivation in host boroughs remained similar and had deteriorated for some measures after the Olympics (12). The continuation of health inequality and housing exclusion is reflected in the impact of the COVID-19 pandemic, as Public Health England (13, 14) reported that ethnic minority communities such as Bangladeshis and Black African Caribbeans are at higher risk of COVID-19 related mortality and morbidity in comparison to White groups. Within the East London context, Newham, Tower Hamlets and Hackney are amongst the worst affected boroughs during the pandemic – all of the boroughs hosted the 2012 Olympics and were rebranded as growth boroughs in 2008 for their potential to enhance investment beyond central London. Based on empirical findings from a research project that aimed to understand the short-term impact of the Olympic regeneration process prior to the COVID-19 pandemic and underpinned by the disproportionate COVID-19 impact on ethnic minority groups, the author argues that the Olympic regeneration did not really bring the expected benefits to the longstanding ethnic minority population of Commonwealth ancestry. The legacy promises and its derivative planning framework had deviated from its initial promise of benefitting these disadvantaged minoritised communities in order to retain interests and prominence of the powerful elites and political expediency (See Figure 1).

Supported by the evidence from sixty in-depth interviews from two casestudy wards (i.e., Bromley-by-Bow and Hackney) which were conducted and analysed during pre-pandemic time, and applying Lefebvre's “right to the city” perspective, the paper reflects that the planning and implementation of the regeneration projects did a little to understand the real and organic needs of the communities, leaving minoritised ethnic communities in a more excluded state. The empirical evidence suggests that the pre-existing minoritised communities are prone to involuntary moving out from the “growth boroughs” because of the increased living costs and non-availability of the promised high-paid jobs for the local residents. The research findings provide the basis for the argument that the games-led regeneration is contributing to an unjust trade-off between “deprived” pre-existing residents and “upper class” gentrifiers. The paper argues that the disproportionate and racialised impact of the COVID-19 pandemic on the minoritised ethnic communities is a vivid example of how inequalities persist and manifest in new ways if *de facto* rights and needs are sidelined, affecting the overall well-being of these historically deprived communities in East London.

The following two sections of the article will give an overview of the disproportionate COVID-19 impact in the UK and the Olympic 2012 legacy-led regeneration process in East London, respectively. Section 3 will summarise the methodology and right to the city theoretical framework in which the fieldwork analysis was embedded. Section 4 will summarise the research findings followed by a concluding section to compare the disproportionate COVID-19 impact as a *de facto* legacy of exclusion and inequality on racialised minority communities. The concluding section also suggests some recommendations to minimise the gap between the rhetoric and what is happening on the ground with a view to promoting the inclusion of the minoritised ethnic communities in the remainder of the delivery of the legacy promise of “transforming the heart of East London”.

## 2. Disproportionate COVID-19 impact: the legacy of spatial and racialised inequality

The higher mortality rate amongst ethnic minority communities was first reported in *The Guardian* newspaper and later supported by many other organisations, such as Public Health England (PHE), Runnymede Trust, and the Office for National Statistics, based on the evidence collated from disproportionate infection, hospitalisation, and deaths rates during the first wave. PHE (13) acknowledged that the pandemic had exacerbated the longstanding inequalities affecting ethnic minorities as they were more exposed for their living and working conditions. The history of ethnicity-related inequality dates back to the 1950s, when immigrants from the new Commonwealth countries started to come in waves shortly after World War II, first Caribbean, followed by immigrants from Indians and Pakistanis and later Bangladeshis (6). A significant proportion of these earliest migrants took jobs in larger cities and tended to settle in areas with weak net in-migration from the White population (1). Towards the end of the twentieth century, the effects of deindustrialisation had increased unemployment and deprivations in the areas with higher concentrations of ethnic minority residents. Moreover, the restrictive immigration legislation predominantly affected the Black and Asian immigrants, and racialised socio-political system gradually paved the way towards contemporary social exclusion of these racialised communities (15). One of the earliest records of health inequality, the 1980's Black Report, presented a hypothesis that race was one of the crucial dimensions of inequality in Britain at that time, as immigrants from New Commonwealth countries had been facing greater difficulty in finding work and adequate housing, but it was difficult to statistically prove the hypothesis because of lack of data at that time. Later in 2010, the Marmot review neglected the issues related to ethnicity-based health inequality, which prompted the government to launch the Public Health Outcomes Framework to assess health outcomes without focusing on ethnicity (9). Therefore, ethnicity remained a neglected parameter for achieving equality through health policy interventions, as they either considered ethnic differences as an outcome of class inequalities or perceived ethnicity as exceptional genetic or cultural factors that drive differences in health experience (9, 16).

**Figure 2:**
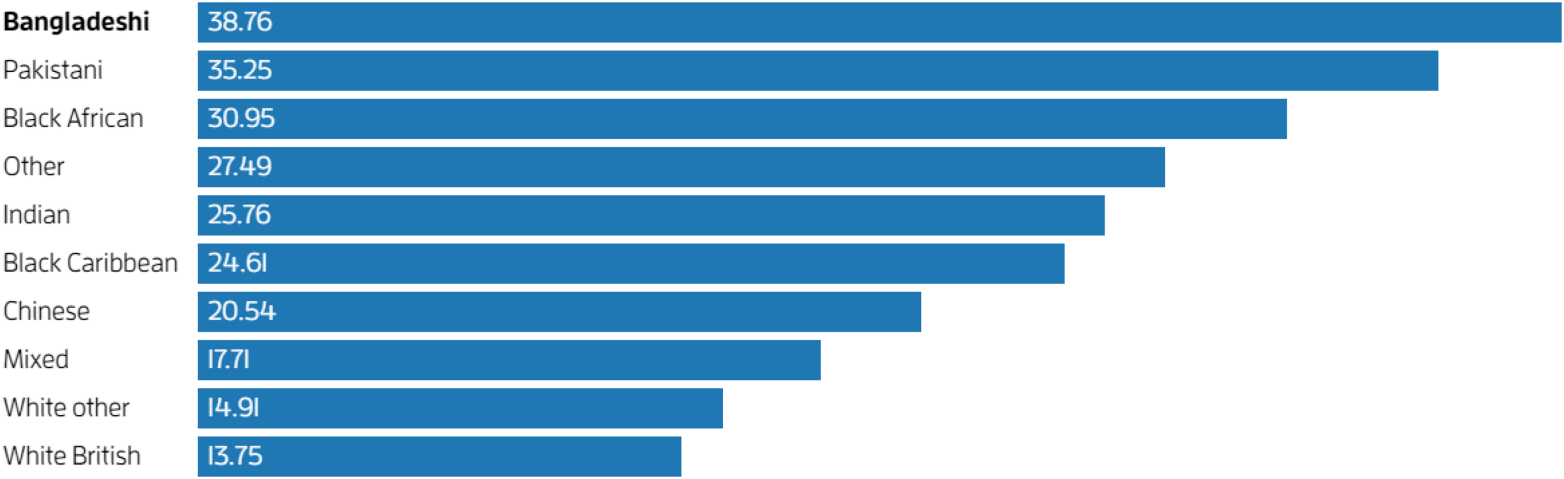
% death (30–100 years) involving COVID-19 in England between 24 January 2020 and 1 December 2021. Source: Murugesu (17).

PHE (14) reported in August 2020 that after accounting for the effect of sex, age, deprivation and region, people of Bangladeshi ethnicity had around twice the risk of COVID-19 related death compared to people of White British ethnicity. The report added that people of Chinese, Indian, Pakistani, Black African and Caribbean ethnicity had between 10% and 50% higher risk of COVID-19 related death when compared to White British. During the second pandemic wave, Bangladeshi, Pakistani, Black Caribbean and Black African people remained at higher risk of mortality than White British people (17). In particular, Bangladeshi men and women were respectively five times and over four times more likely to die in comparison to male and females of the White British groups. Office for National Statistics (18) reported that based on the number of people between the ages of 30 and 100 who died between January 24, 2020 and December 1, 2021 in England, COVID-19 was involved in a higher proportion of deaths in all ethnic minority groups than in White British people. Figure 2 presents an analysis from New Scientist^2^ (17) suggesting that people of Bangladeshi descent were worst affected as COVID-19 was involved in 39% of their deaths, followed by Pakistani descent (35%), Black African descent (31%). Moreover, since Omicron became the main variant, Bangladeshi males have had the highest rate of death involving COVID-19, 2.7 times higher than males in the White British ethnic group; this was followed by Pakistani males (2.2 times) and Black Caribbean males (1.6 times)^3^.

Many scholars believe that pre-existing inequalities and disadvantages of ethnic minority communities were likely to have contributed to the disproportionate impact of the COVID-19 pandemic on Black African, Black Caribbean, Bangladeshi, Pakistani and, to a slightly lesser extent to Indian minority group (16, 19). Multiple factors have been articulated in explaining the disproportionate health outcomes during the pandemic, such as concentration in urban areas, living in overcrowded and substandard households in deprived areas, having low income and savings, working in jobs with higher expose to infectious diseases, comorbidities and cultural factors such as multi-generational households etc. (4, 20). A significant proportion of Bangladeshi and Black African Caribbean people live in Olympic host boroughs, nearly a fifth of the national total of British-Bangladeshis lives in Tower Hamlets borough (21). In addition to that, throughout the pandemic, lack of digital literacy and the pre-existing “digital divide” has hindered access to governments' online-based services and exacerbated the sufferings of a section of those ethnic minority groups (16, 22). Hence, the disproportionate impact of COVID-19 on ethnic minority groups throughout the pandemic is the continuation and manifestation of longstanding socio-spatial inequalities which sparked racial inequality debates in the UK (23).

At the territorial scale, infection rates have been the highest in urban areas, and thereby, ethnic minority populations are more impacted as they tend to live in cities. For instance, the majority of the Pakistani (99.1%), Bangladeshi (98.7%), and Black African (98.2%) population of the UK live in cities. In fact, 58.4% of Black people and 35.9% of Asian people in England and Wales, live in London, the city which was hit hardest with a higher number of confirmed cases in England during the first wave. London's ethnic minority groups have been concentrated in inner boroughs in East London, such as Newham, Tower Hamlets and Hackney which were classed as most deprived according to the Index of Multiple deprivation 2010 and 2015. PHE (14) reported that “the impact of COVID-19 has replicated existing health inequalities and, in some cases, has increased them” because the COVID-19 mortality rates were more than double in the most deprived areas than the least deprived areas. ONS's figure showed that in England, Black and Asian people are more likely to live in the 10% of most deprived neighbourhoods inclusive of the Olympic host boroughs. An analysis from ONS^4^ found that between 1 March and 17 April 2020, all London boroughs had the highest age-standardised death rates compared to other regions, where Newham has had the highest age-standardised rate with 144.3 deaths per 100,000 population in London. So, Newham was one of the most affected boroughs from 1 March to 17 April 2020 while other boroughs like Hackney and Tower Hamlets also had a higher death rate.

Considering East London's sufferings throughout the pandemic intertwined with evidence of the disproportionate COVID-19 impact on ethnic minority communities nationwide, it could be argued that the pandemic has clearly highlighted the legacy of racialised inequality, particularly in the context of Olympic 2012 host boroughs. In the past, there had been a number of attempts to regenerate parts of East London, such as the Docklands Development which led to further exclusion of the local residents (24). The legacy-led regeneration plan has been portrayed as a continuation of spatial regeneration in East London to minimise deprivation gaps with the rest of London. The legacy projects set out to transform the communities through regeneration momentum by 2031, but before finishing a decade of regeneration, the uneven impact of COVID-19 pandemic on the ethnic minority groups demonstrated that minoritised ethnic communities currently remain severely deprived in terms of health, housing and employment provision.

## 3. The legacy 2012: An attempt to build the social-economic and physical infrastructure of the area

The provision of a social legacy was a core component of London's bidding document, stating that “the most enduring legacy of the Olympics will be the regeneration of an entire community for the direct benefit of everyone who lives there” (25). After winning the bid, the legacy-led regeneration process was implemented by different short-lived and expert-led organisations, such as ODA (i.e., Olympic Delivery Authority, responsible for spatial transformation of the park area) and LOCOG (London Organising Committee of Olympic Games, officially designated as the organiser of the Olympic Games). However, due to the global recession in 2007 and the subsequent emergence of austerity measures in 2008, the social legacy was undermined by the economic regeneration by ODA and LOCOG (5, 26), and rebranded as “regenerating the heart of East London” (DCMS, p. 6) in 2007. The intended regeneration efforts were further strategised as *transforming the space* (i.e., by transforming 250 hectares of wasteland into the Olympic Park), *Transforming Communities* (i.e., by promoting social cohesion and integration within local communities) and *Transforming Prospects* (i.e., by creating 12,000 new jobs in the area of the Park post-Games). In addition, there was a promise for employment legacy, which may have prompted low-paid jobs to locals but lacked genuine and solid strategies to enable local people to get high-paid and sustainable jobs (5). Indeed, achieving social legacy by minimising deprivations across East London is sidelined to prioritise the economic regeneration by adopting an undemocratic legacy framework and its exclusionary planning rhetoric throughout the regeneration process.

East London's inherent cultural diversity and ethnic clustering were showcased and celebrated during the hosting of the Olympic event. The Olympic host boroughs have distinct characteristics in relation to their ethnic minority populations. For example, Tower Hamlets and Hackney have relatively higher proportions of Bangladeshi and Black African Caribbean residents compared to London as a whole [see Ref. (24)]. Regarding the clustering pattern, Peach (27) observed that the Bangladeshis were blue-collared and council housed in the inner city (in terraced and flatted properties), mostly in Tower Hamlets and Spitalfields, while the Caribbeans were also blue-collared with substantial representation in council housing but far less segregated than the Bangladeshis. In the legacy promises, the ethnic minority communities were the *de facto* targeted groups as they were adversely affected by the deindustrialisation process in terms of material poverty, unemployment, and socio-economic deprivation while living in crowded or sub-standard households. During the early years of the regeneration scheme, the legacy process emphasised physical transformation in terms of renewing the urban fabric and housing stock, while the holistic socio-economic themes received less attention and were superseded and sidelined in various ways. After the Olympics Games, ODA and LOCOG were dissolved, and the Mayor of London established the London Legacy Development Corporation (LLDC) to implement legacy-led regeneration projects in the park and its adjacent areas. LLDC published the Local Plan in 2014 to transform an area covering 480 hectares inclusive of the Olympic Park and an additional 253.4 hectares of land from the host boroughs to implement regeneration projects until 2031. After the Olympics Games, the legacy regeneration projects was confined within the boundary of the Legacy Corporation while the six host boroughs were expected to utilise the newly transformed Olympic Park to boost their socio-economic inclusion in the name of the “convergence” agenda. In addition, host boroughs also have their own Master Plans shadowing the London Plan 2004.

Ziakas (28) and Chalip (29) reflected that the efficacy of the economic impact depends on host communities' capability in which benefits could be harnessed and nurtured from its common resource base within a temporally limited set of opportunities. In London 2012 context, the adopted legacy framework has largely failed to harness and foster the desired long-term socio-economic impacts since the needs and desires of the disadvantaged ethnic communities were traded off to serve the interests of powerful elites, event owners, and political expediency. The east end of London still accommodates a concentration of ethno-cultural communities which also displays signs of socio-spatial inequality along ethnic lines amidst the power and wealth of the imperial capital. The host boroughs like Tower Hamlets, Newham and Hackney remained amongst the most deprived according to Index of Multiple Deprivation 2015 (IMD2015) and residents had been experiencing poverty and exclusions amid the dynamism of legacy-led transformation changes. Since the bidding phase, the policy rhetoric had spoken about the socio-economic inclusion of disadvantaged residents. However, the disproportionate COVID-19 impact reflected that such inclusion is yet to be achieved even a decade has passed since the 2012 Olympic Games. So, although Olympic urbanisation is capable of dramatically reforming the local built environment, the success of regeneration needs to be understood in terms of how the initiatives have reduced the entrenched and persistent inequalities by providing decent housing, high-paid jobs and better opportunities to host population. For lower-income East Londoners living in host boroughs, the Games effects resonate with presumed displacement, either directly via housing demolitions, landlord evictions and rent increases, or involuntary eviction to the more deprived areas (30). So, despite the broader vision for converting the Olympic sporting heritage into residential and community services, the extent to which the local minoritised ethnic communities have benefitted from the legacy-led transformation is questionable.

## 4. The research methodology and theoretical framework

Purcell (31) and Harvey (32) have argued that the rights-based approach can be proactively used to give voice to the voiceless who are often left out for being in the bottom layer of our unequal societies (i.e., the so-called lower class of Marx's class system). Lefebvre's right to the city entails that those who inhabit the city, irrespective of their social class, must reclaim the city in order to carry forward their claims to inhabit urban space well (31). Therefore, the theoretical framework of the research project from which the findings are drawn, adopted Lefebvre's (33) concept of the “right to the city” with a view to exploring ethnic minority communities' “de facto” rights within the initial impact of the hegemonic legacy regeneration projects. The empirical work of the research was embedded in Purcell's (34) two principal rights, viz: “right to participation” and “right to appropriation”. On the one hand, analysis through the “right to participation” concept helped to understand the barriers that have restricted ethnic minority communities' meaningful participation and empowerment in decision-making processes and on the other hand, applying the “right to appropriation” concept highlighted their de-facto needs and frustrations when appropriating legacy-led transformative changes. In this way, the right to the city approach provides a holistic and inclusive framework to understand the minoritised communities' lived realities in adjusting to the legacy-led socio-spatial changes in East London.

**Figure 3:**
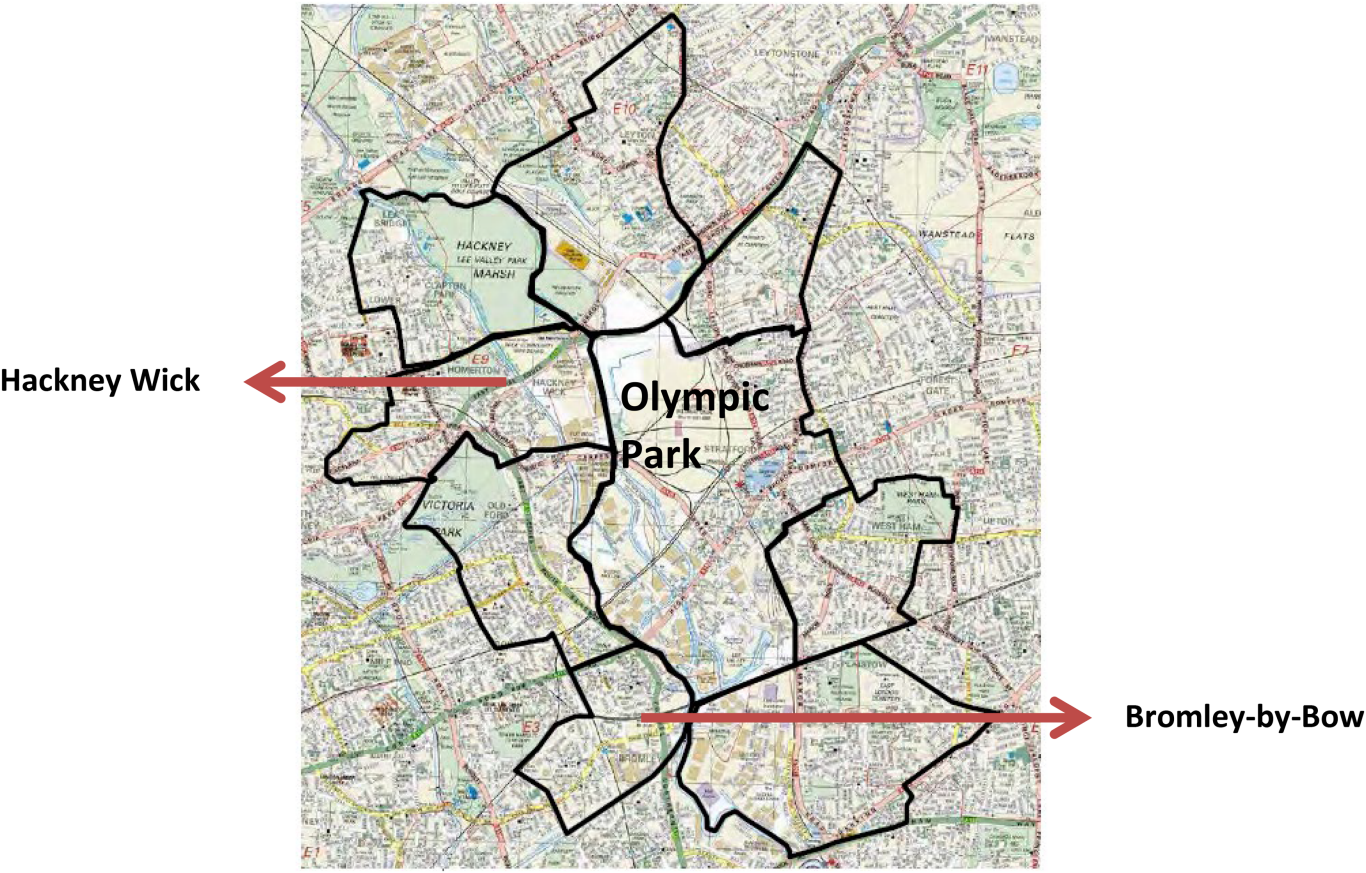
Olympic park and case study wards. Source: Reproduced from a figure in LLDC's *Community Engagement Policy*, (2012, p. 12).

To explore residents' lived realities in the dynamic legacy 2012 transformative process, two adjacent wards of the Olympic Park area, namely Hackney Wick (HW) and Bromley-by-Bow (BbB), were purposively chosen from Tower Hamlets and Hackney boroughs, respectively (See Figure 3 for the location of the case study wards). These two wards also fall amongst the 5% of the most deprived wards according to the Index of Multiple Deprivation 2010 (IMD2010), where HW ranked 5 and Bromley-by-Bow ranked 12 (i.e., the average score of deprivation within London where 1 = most deprived and 628 = least deprived). Lower deprivation scores in the IMD2010 suggested that the residents of those wards were experiencing higher levels of deprivation within London according to the seven distinct domains (i.e., income, employment, health, education, housing and services, living environment and crime). In addition to that, both HW and BbB wards share some of their areas with Legacy Corporation and have a higher concentration of ethnic minority communities. For instance, according to the 2011 census, 70.4% of residents in BbB were from non-White by ethnicity, and most non-White residents belonged to the British-Bangladeshi ethnicity (44.9%). In addition, 51.6% of residents in HW were non-White, while the Black (African-Caribbean and other Black) ethnic group altogether comprise the largest ethnic minority group (31.3%) according to the census 2011.

The research methodology was based on qualitative methods (i.e., semi-structured interviews, photo-elicitation interviews, and the researcher's direct and unobtrusive observations) to gather empirical evidence. In total, sixty in-depth interviews were conducted through purposive sampling from March to June 2015, of which 78% of interviewees are from the Black and Asian ethnic minority communities and the rest from mainstream White-British, White-Irish, White-German and mixed ethnic backgrounds. Almost all of the interviews were conducted in the interviewees' residences, and a few have been conducted in public places (e.g., local parks & cafés where data protection rules were strictly followed) within an agreed timeslot of an hour. Most of the interviews were conducted in English, and some in BbB were conducted in Bengali. The highest percentage of interviewees were aged between 36 and 40, and around one-third of the interviewees lived in council housing. When comparing interviewees' responses with the National Readership Survey's Social grade, more than half of the interviewees were categorised as “working class”, which helped to visualise a broader picture of the socio-demographic characteristics of the fieldwork interviewees.

Moreover, twelve of these interviewees also took part in a photo-elicitation interviewing, where they were asked to photograph their local places, activities or physical changes which they experienced as problematic and perspective in relation to the adjacent Olympic Park and subsequent legacy-led changes. Some people photographed the Olympic Park area, construction sites and local businesses; some photos captured specific local problems in terms of housing situation, parking provision, garbage disposal, disturbance from construction debris and so on. In addition to the residents, relevant professionals and experts (such as local councillors, ex-councillors, officials from local NGO & housing associations, and social workers) were interviewed to get expert opinions on how the regeneration process was affecting the lives of ethnic minority residents. Table 1 provides the ethnic backgrounds of interviewees as well as the number of key-informants from each ward who took part in the research project.

**Table 1:** Number of interviewees and key informants.

Ward	Interviewees			Key informants
	Non-White ethnicity		White-British, White-Irish, White-European & mixed ethnicity	(Councillor/ex-councillor/officials of council & NGOs)
	Bangladeshi	Black African-Caribbean		
Bromley-by-Bow	25	0	5	9
Hackney Wick	0	22	8	4
Total	47		13	13

The analysis of the field data was conducted applying Purcell's (34) notions of “right to participation” (i.e., problems and prospects concerning residents' participation in the planning process) and “right to appropriation” (i.e., appropriation of legacy-led transformed spaces), underpinned by Lefebvre's (33) “right to the city’s vision. All interviews were transcribed in English and were initially coded in NVivo according to six emerging themes (e.g., participation before the Olympics, participation after the Olympics, legacy-related benefits, legacy-related problems, access and use of transformed spaces, ethnic integration and segregation). The information from the photo-elicitation interviews and key-informant interviews were also coded according to the emerging themes. As analysis progressed, the emerging themes were broken down into more specific themes to regenerate findings under the two broad themes, viz: right to participation and right to appropriation. Data were anonymised and interviewees” real names were masked with fictitious names.

There were scopes for triangulation of the research findings since London Olympics and regeneration projects were the loci of a handful of research projects and urban scholarships. The empirical findings are resonated with Watt's (30) reflection on legacy-led displacement and spatial inequality, Bernstock's (35) view on inclusive regeneration, Marrero-Guillamón's (36) observation on public participatory mechanisms and Vadiati's (5) evidence on unsustainable and low-paid jobs for the locals in the name of employment legacy. Drawn from sixty local residents' lived experiences, this finding provides nuances in terms of how the desires and needs of the minoritised communities' were adjusted and compromised in the process of hegemonic and top-down legacy-led socio-spatial changes.

## 5. Transforming the heart of east London: legacy rhetoric vs. residents' lived experience

Lefebvre's right to the city entails that those who inhabit the city, irrespective of their social class, must reclaim the city in order to carry forward their claims to inhabit urban space well (31). Drawn from sixty local residents’ lived experiences, this section provides a discussion of the research findings under two broad themes, viz: “right to participation” (e.g., residents' central role in decision-making processes) and “right to appropriation” (e.g., access and use of urban spaces).

### 5.1. Right to participation: the systematic disempowerment of ethnic minority residents

In a broader sense, the empirical evidence drawn from the research works suggests, local ethnic minority residents’ participation was tokenistic, as such they had access to planning-related events, but in reality, they could not make any impact to integrate their needs and aspirations at different stages of the legacy-led regeneration process. The literature and empirical findings suggested that there are some pre-existing barriers (e.g., the irregular and narrow window of direct participation, one-way flow of information from Olympic Games promoters to residents etc) which became more intense during the spatial transformation of the Olympic Park area and thereby, excluded the local ethnic communities from meaningfully participating in the planning and decision-making processes. The core problems causing the exclusion and disempowerment of ethnic minority residents in the planning and decision-making process were rooted in the systemic disempowerment of the minoritised ethnic communities stemming from the chronic socio-structural inequalities in East London. Such exclusions were further reinforced by the Olympic-led top-down pressures, which had contributed tokenistic participation of minoritised ethnic communities living in the host borough areas and systemically kept them disempowered and disengaged in local planning, decision making and project implementation processes. Drawing on the literature review and empirical findings, the core problems which have hindered ethnic minority residents from taking a direct and central role in planning and decision-making processes are discussed as follows:

#### 5.1.1. Limited room for direct participation during both pre-Olympic and post-Olympic planning and implementation processes

The interview analysis suggests that top-down planning and implementation process principally responded to neoliberal market forces, where the minoritised ethnic residents became disempowered and marginalised in many ways. The fixed dates of the Olympic Games acted as a catalyst to materialise the speedy physical transformation of the new park, and therefore, after winning the bid, direct citizens' involvement remained nominal in the formulation of the Legacy Master Plan 2007 by ODA, which was the blueprint for timely deliverance of the Olympic Park and complementary facilities. As the importance of public involvement was mentioned in some Olympic planning documents, so, before the Olympics a relatively tight window of public participation was arranged for those who were affected by the land allocation process (37). Therefore, the physical transformations were undertaken in a top-down fashion where the voice of the local residents was scarcely heard.

After the Olympics, the national interest of legacy-led regeneration remained top-down and undemocratic, as Marrero-Guillamón (36) observed that public hearings to finalise the LLDC's Local Plan (2015–2031) were highly tactical and expert-led, where ordinary residents remained disempowered. Interview analysis and literature exploration suggest that some efforts to consult locals were made but in reality, information regarding consultation events reached only a fraction of people who had the skills (e.g., good English, knowledge of using the internet and online services and so on) to navigate planning-related matters. For example, Mr. Anderson (a Black-African gentleman in HW) said:

*“The plans were there to follow the desire of the elected people and those projects will be completed in no time to benefit the people who were in politics.”*

In a way, both before and after the Olympic Games, the major actors (e.g., ODA, LLDC) and the councils were more interested in ensuring public engagement to justify the land readjustment process (through the Compulsory Purchase Order) but not in how these acquired lands could be used and transformed through deliberate participation of the residents in a democratic way.

#### 5.1.2. Exclusion of minoritised ethnic communities due to nominal participation of the elected representatives in the local planning process

The councillors of the host boroughs act as advocates for the locals through representative democracy in urban affairs. However, the existing form of representative democracy was not enough to include poor, marginalised and hard to reach residents as the elected people represent the majority but not necessarily the ethnic minority residents. Two key-informants in this research reported that, both before and after the Olympic Games, one nominated councillor from each host borough participated in the ODA and LLDC's meetings, respectively. So, in those meetings the selected councillors were representing hundreds of thousands of borough residents as boroughs like Tower Hamlets and Hackney had a population of 254,100 and 246,300 respectively (census 2011).

The key-informats also reported that not all councillors received nominations to attend the ODA and LLDC meetings and the nominated councillor from the borough hardly got an opportunity to represent residents' diverse views including the needs of the minoritised ethnic communities. Empirical evidence suggested that during the post-Olympic years, some councillors felt disempowered as they could not participate in LLDC meetings unless the Mayor (of host boroughs) nominated them. For instance, a key informant (i.e., ward councillor) reflected:

*“The Mayor decides which councillor will represent the borough in the LLDC's meeting. So, I could not go to the LLDC's planning meetings unless the mayor nominates me. Ward councillors like me feel disempowered in this way”*.

During the fieldwork, the author also observed that the LLDC was materialising physical transformation of the Olympic Park and its surrounding area using all its power and resources while participation even from the elected representatives was often sidelined for the sake of speedy delivery of post-Olympic projects.

#### 5.1.3. Minoritised residents' lack of confidence and motivation to influence the top-down decision-making process

Citizens are expected to be knowledgeable to promote their interests more effectively in public events and decision-making meetings; however, the interview analysis suggests that minoritised ethnic communities bordering the Olympic Park area lack confidence, motivation and commitment to influence top-down decision-making processes because of language-related shortcomings and lack of previous experience of participatory democracy in their country of origin. The field study findings found that a small percentage of local interviewees participated in the consultation process, where no special efforts had been made to directly hear “the hard to reach” and disadvantaged ethnic minority residents. In addition, a few interviewees from a White-British/European background who participated in post-Olympic planning events could not recall seeing any significant attendance from ethnic minority groups despite the fact that more than 42% of the population of the Legacy Corporation area and its adjacent boroughs (i.e., Hackney, Newham, Tower Hamlets and Waltham forest) belonged to an ethnic minority background (10). Mr. Becker (a member of the Artist community from White-European background) reflected:

*“I have participated in most of the LLDC's planning events. I was interested because the plan will affect the people from the Artist community in the Hackney area. But I could not recall seeing any Asian or Black people in those meetings. The participants were mostly White at a glance.”*

Moreover, fieldwork analysis suggested that ethnic minority residents are not well-organised and there is no unified voice from them to challenge undemocratic planning decisions at the neighbourhood level. Though several NGOs and youth groups (e.g., Bromley by Bow Centre, Artist community in Hackney Wick) had been working for the residents' welfare, minoritised communities are yet to be mobilised to influence and challenge the local decision-making processes for a resident-led alternative future.

#### 5.1.4. Language barrier and digital illiteracy excludes people from minoritised communities

The field study evidence suggests that local residents were communicated through letters and notices via communal noticeboards for public meetings, consultations or development works. Interviewees reported that most of these notices were just to inform them about activities that would affect their everyday life (e.g., parking restrictions, etc.). Some residents reflected that the notices and letters were all about the decisions rather than invitations to get involved. The flow of information had predominantly followed a one-way route (i.e., council/LLDC to residents) as interviewees who wrote to councils regarding local problems did not get any reply in most cases. Moreover, due to language barriers, a few Bangladeshi female interviewees reflected that they could not read the letters and notices written in English.

Moreover, the literature review suggests that more recently, public concerns are widely communicated via council and LLDC websites. For instance, residents were encouraged to respond online during the consultation process for the LLDC's Local Plan [see Ref. (38)]. Though the planning systems of the developed countries are increasingly using electronic provision (39), studies show that those who suffer deep social disadvantage are more likely to be disengaged from the internet than those who are socially advantaged (40). So, many members of the ethnic minority residents remained *de facto* excluded from the LLDC's participatory mechanism as the alternatives to overcome the technical or language-related difficulties were not easily available to them.

In addition to that, Marrero-Guillamón (36) observed the public hearing sessions on LLDC's Draft Local Plan in March 2015 and he reflected the participatory mechanism remained exclusionary for ordinary residents along class, gender and ethnic lines and only the well-educated professionals (e.g., architects, lawyers, or urban studies scholars) could contribute to the planning process as they had the skills to navigate legislative and planning related matters. He wrote:

*“Once on the table, successful ‘participation’ depended on being able to understand and deploy a highly technical language. Indeed, most of the people around the table had some kind of training that facilitated this: lawyers and planning consultants in the case of the developers and the LLDC; architects, community organisers or urban studies scholars in the case of the community groups”* (Marrero-Guillamón (36), p-226)

So. following Marrero-Guillamón's (36) observations, it could be argued that the minoritised ethnic communities were prone to be more excluded in the planning process as some of them were not good at English language and e-communication, which kept them segregated from wider use of online products and services.

Therefore, the empirical research found that ordinary ethnic minority residents from neighbouring wards of the Olympic Park remained largely powerless and excluded from the post-Olympic planning and decision-making process in many ways. Such language-related barriers and digital illiteracy might have contributed to disproportionate COVID-19 impacts on minoritised communities since people were forced to adapt remote care and online-based services, due to having limited or no other options available (16, 22).

### 5.2. Right to appropriation: ethnic minority residents' adjustment to the legacy-led changes

Purcell (31) and Harvey (32) have argued that the rights-based approach could lead towards a proactive way to give voice to the voiceless, such as the working-class communities, for producing and appropriating urban space well (34). Therefore, within a capitalist and a free market economy, right to appropriation can confront capital's ability to control urban space, resisting the current hegemony of property rights and stressing the primacy of the use rights of inhabitants (34). The interview analysis underpinned by Purcell's (34) notion of right to appropriation reflected that the tangible benefits residents received before the Olympic Games include a patchwork of cleaned up outdoor and vacant spaces along with additional lighting and security cameras which made the area look better. However, these benefits were small compared to the problems they encountered, some of these problems are discussed below.

#### 5.2.1. Increased living cost and fear of involuntary displacement

The literature review suggests that to make the way for the new park, a number of residents including Gypsies/Travellers and small businesses were evicted by dint of compulsory purchase orders (37). In addition, some pre-existing residents also felt frustrated because the combined regeneration efforts of the LLDC and the councils have increased the living cost in the area, for instance, Mr. Fleming (a Black African gentleman) reflected that:

*“Olympics did not benefit me at all, instead it increased my living cost. The on-street parking was free before the Olympics but now I have to pay an annual charge to park my car in the road*.

*Council started on-street parking charges just before the Olympic Games; people thought it was for easing traffic during the event. But it did not go away after the Games. Now Hackney residents have to pay an annual charge for on-street parking.”*

Most interviewees were frustrated as they feared that they might need to leave the area to contain their living expenses in the near future. Interviewees who were living in rented accommodation, with or without getting housing benefits, expressed their fear of involuntary displacement. The private tenants who received housing benefits expressed disappointment because they were paying additional money on top of housing benefits to fill the rent gap. Ms. Hasan (a Bangladeshi lady in BbB) said:

*“Now we are living with my in-laws in a council flat. We want to buy a house nearby, but I could not afford a house here in Bromley-by-Bow now. We don’t want to leave the area as my parents and relatives live here, may be in near future we need to move further East, somewhere in Essex, because we can’t afford the higher rent or higher mortgage payment in Bow area.”*

Residents lived experiences reflected that the modern infrastructure and security provision in their area were inviting outsiders from more affluent classes to move in, threatening the security of tenure of the existing tenants.

#### 5.2.2. Gentrifications and disappearances of socialisation places

In recent years, Tower Hamlets borough constructed a number of new buildings in BbB and a few sites were under construction during field investigation, which would increase the housing stock in the area. However, the empirical findings indicate that the new build housing estates have a few council tenants as a major portion of the housing were sold off to the upper-class newcomers. Although some Bangladeshi people were positive, as they thought new buildings would benefit those who were on a long waiting list for getting council accommodation, some others felt more housing needs to be allocated to longstanding minoritised ethnic residents who are living in crowded and unhealthy accommodations. In general, most interviewees reflected that their areas were inviting gentrification because of new-built apartment blocks and improved public transport services. Besides, the newly installed security provision (e.g., surveillance cameras) were also viewed as a mixed Olympic blessing as some people said those security instalments were communicating the impression of a safely built space and positively motivating newcomers to live in the case study wards. The findings are resonated with Watt's (30) and Broughton's (41) findings which argued that the regeneration projects had reduced the number of socially rented houses and invited gentrifiers to inhabit in new-built houses ignoring the organic need of the pre-existing residents.

**Figure 4:**
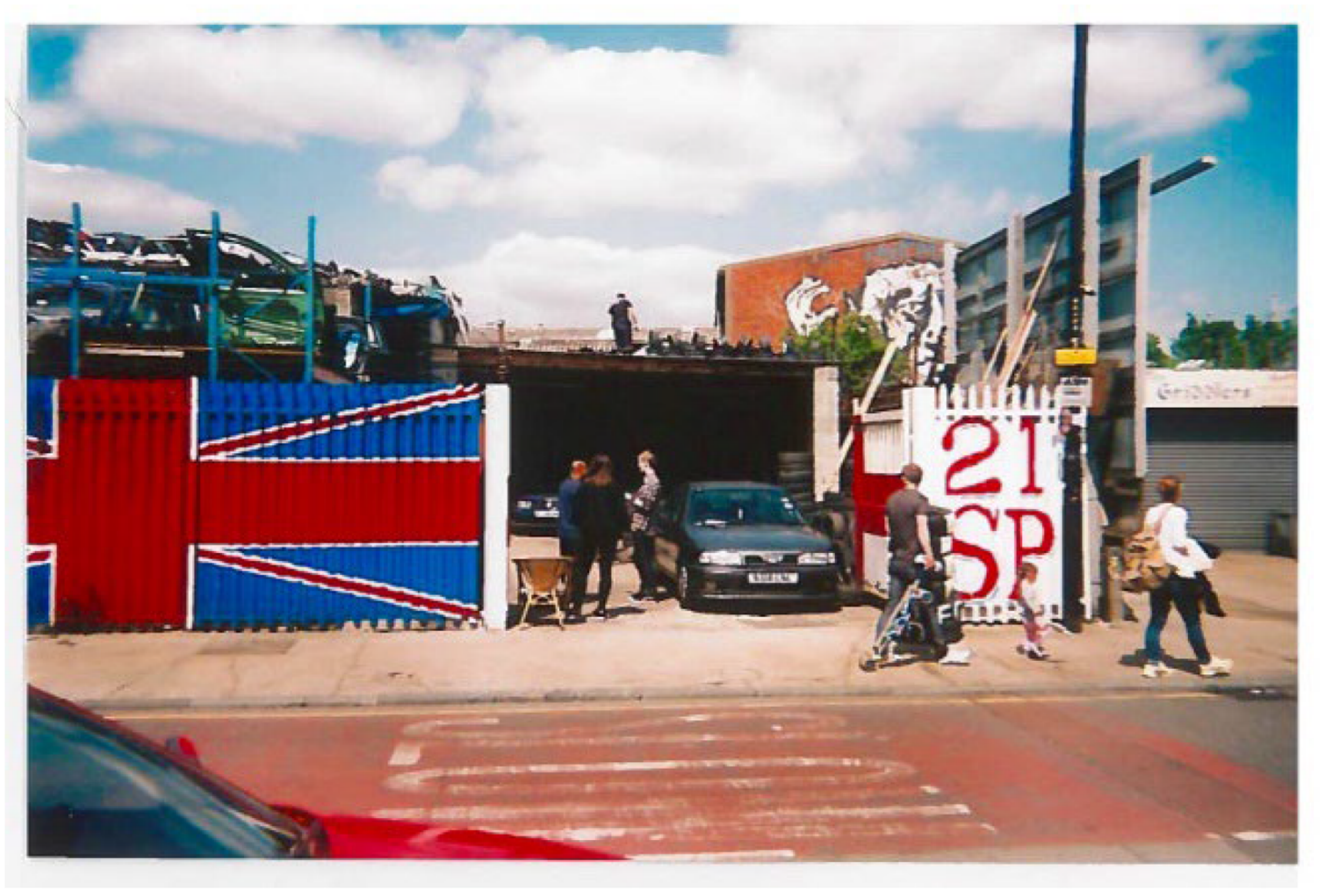
Gentrifications and disappearances of socialisation places. “*A car mechanic's shop owned by a Bangladeshi man; this shop might be gone in the next few years.”* (Photo-elicitation interview with Mr. Becker, an artist from White-German background).

In addition, a few HW residents were disappointed as their usual ethno-cultural communal facilities (e.g., shops, pubs) were slowly disappearing in the area, so they had to travel to other places to interact with each other. Most of the interviewed HW residents, including members of the artists' community, felt that the pockets of vacant land would disappear soon while some other land uses (e.g., motor workshops, see Figure 4) were anticipated to be converted into new apartment blocks inviting gentrification.

Some residents who were born and raised in HW, felt that the area was changing very fast, and the suburban calm and quietness were being replaced due to processes of neo-liberal modernisation. Frustratingly, the pre-existing residents could not stop these state- and market-led transformations but had to adjust if they wanted to continue to live in the area.

#### 5.2.3. Legacy did not deliver promised benefit to local businesses

The BbB area is physically divided by the A12 highway into east and west, where the eastern segment of BbB had fallen under LLDC jurisdiction but not the west, so the LLDC has limited involvement in the western part of BbB. The pre-Olympic document “*Stitching the fringe-working around the Olympic Park”* stated that a new town centre would be transformed in the east of A12 but the legacy authority could not build the centre because of legal disputes with the landlords (42). Later, the previously proposed physical transformations to the east of the A12 were dropped in the LLDC's Local Plan. Moreover, some local entrepreneurs and business owners in BbB felt betrayed as some promised redevelopment works of the marketplace did not go ahead as planned. Ms. Dipa (an entrepreneur) said:

*“We (entrepreneurs and shop owners in Stroudley Walk marketplace) attended a series of meetings with planning officials of the Tower Hamlet borough and Poplar Harca staff before the games, we thought our business will boom with the Olympics, but nothing improved, we gained nothing.”*

Besides, the local entrepreneurs and shop owners had to pay more rent and council tax compared to the pre-Olympic years while economic returns from businesses remained the same during the post-Olympic years.

#### 5.2.4. Legacy did not deliver sustainable & well-paid jobs to local residents

The government promised to tackle decades of exclusion by providing better career prospects through the social legacy, especially for young people (43). However, one of the key frustrations of the ethnic minority residents was the non-availability of these opportunities. Most of the young interviewees expressed disappointment as the Olympic efforts did not offer them sustainable and well-paid jobs. Mr Blair (a Black Caribbean gentleman) said:

*“Lots of people thought the Olympics would lead to well-paid jobs and many youngsters like me visited the recruitment agencies for work. Though I secured a short-term security job during the Olympic time and the payment was good then, but later had to leave the job because of the reduced hourly wage-rate”*

After the Olympics, a few BbB residents were offered retail or low-skilled jobs which led to frustration. So, the people who expected to get better jobs created by the Olympic efforts were disappointed as that did not happen in reality. Local people's dissatisfaction on not getting high-paid jobs and being only considered for low-skilled jobs has also emerged in Vadiati' s (5) research where she found an absence of a genuine and solid strategy to help the local residents to secure more highly paid jobs. The young workforce from ethnic minority communities in East London, who are more educated and skilled than their parents and grandparents, felt particularly betrayed as the Olympics made them ambitious about joining the mainstream economy of London.

Moreover, a key informant (i.e., BbB councillor) reflected that people, who lived outside of East London, got construction and other jobs which were meant to appoint local residents. Employers considered “proof of address” as the criterion for being East Londoners and the literature review showed that anyone who could supply proof of residence in East London, could be considered as a local resident to take up the jobs and other priorities reserved for the locals [see Ref. (44)]. Therefore, some people from outside East London had used the address of hostel or temporary accommodation and got the jobs which were prioritised for local residents.

Furthermore, some Bangladeshi interviewees were positive about improvements in local schools that that has brought about by the Olympic-led regeneration as ethnic communities, such as Bangladeshis, consider education as the best vehicle for upward mobilisation for their children (5). Residents reflected that local schools were renovated and provisions for co-curricular activities were increased which would enhance the educational achievements of the local pupils in the future.

By and large, the residents who had volunteered during the Olympic Games and Olympic-related events felt more positive about the transformative changes and some felt positive as East London received attention and funding while some others were disappointed because of the non-delivery of the decent housing, high-paid jobs and other promised opportunities for the local minoritised communities.

## 6. A decade of Olympic 2012 legacy: the *de facto* exclusion and disproportionate COVID-19 impact on minoritised communities

The Olympic-led spatial transformations are distinctive examples of how global influence and national interests can quickly transform the host city's landscapes where poor and disadvantaged local residents are likely to remain on the losing side. Evidence from Barcelona, Seoul, Atlanta and Sydney reveals that Olympic-led urbanisation has derived very different meanings for poor and vulnerable urban dwellers, in some cases leaving the poorest in a more vulnerable condition (45). Powerful elites drive the dynamics behind channelling the power of Olympism into undemocratic physical transformation under the auspices of globalised market forces. The 30th Olympic Games and their legacy-led regeneration were not different though the 2012 Olympic Games promoter had claimed that the Games would help narrow the deprivation gap between the host boroughs and the rest of London (46). However, regenerating the city's industrial skeleton was challenging as the working-class residents had been experiencing multi-dimensional exclusions for decades despite Dockland regeneration projects. The policy framework of Legacy 2012 claimed that the regeneration efforts would promote the inclusion of poor and deprived ethnic minority residents, but in reality they have been producing exclusions for the disadvantaged ethnic minority communities, such as nominal or limited participation in decision making processes and increments of living costs in the host boroughs. Therefore, for lower-income East Londoners from minoritised ethnic backgrounds living in the vicinity of the Olympic Park area, the effects of the Games resonate with potential displacement, either directly via housing demolitions, landlord evictions and rent increases, or indirectly via involuntary displacement (30, 46).

In this research, the application of a rights-based theoretical framework, in which residents should have the “central” role in production of urban spaces, has increased the understanding of problems and barriers (e.g., in relation to participating and appropriating the legacy-led transformed spaces) that hindered residents' participation and appropriation in a top-down sport-led regeneration process. The combined notions of the right to participation (e.g., direct role in decision making systems) and the right to appropriation (e.g., access to and use of services and provision), have provided holistic approaches to promote a broader notion of inclusion and to challenge the prescriptive way in which urban transformations are being implemented. However, the research findings suggest that while the physical transformation of the Olympic Park with sports-focused infrastructure was fast-tracked to host the Olympics Games, the promised socio-structural transformation through prioritising *de facto* rights of ethnic minority communities (e.g., jobs, housing, etc.) were sidelined to support the top-down neoliberal agenda. The rhetoric was that the host boroughs were expected to be regenerated by the “convergence” effect while the local residents would benefit from newly created jobs and housing, but in reality, wards like Hackney Wick and Bromley-by-Bow became desirable places to live attracting new arrivals. Because of improved public transport and other civic facilities. Regarding housing, most of the field interviewees expressed that there should be more housing for the council tenants as a major portion of the housing were sold off to the newcomers with higher socio-economic status. In addition, some local entrepreneurs felt betrayed as the Olympic efforts did not deliver the expected economic returns and young interviewees were disappointed as the legacy did not offer them sustainable and well-paid jobs. Moreover, pre-existing residents expressed fear of involuntary displacement in the near future because the area would become unaffordable for them. The empirical evidence collected through the research project suggests that games-led regeneration in London is perhaps leading to an unjust trade-off between working class and upper class (and middle class) gentrifiers, ignoring the real and organic need of low income minoritised ethnic residents.

Soon after the completion of the research project, the COVID-19 pandemic hit the UK and hospitals were overwhelmed by the higher rate of COVID-related infection and deaths. During the first wave of the pandemic with the unprecedented number of deaths, a myth was initially popularised echoing Michael Gove's comments on 27 March 2020 that “the virus does not discriminate”^5^ rich or poor, powerful or powerless. Soon social media was flooded with criticism about the unequal sufferings the pandemic has brought about to the poorer section of the country, hence Damian Barr tweeted on 20 April 2020 “*We are not all in the same boat. We are all in the same storm. Some are on super-yachts. Some have just the one oar”*, referring to facts that the pandemic was not an equaliser as there was disparity in living conditions, such as living in crowded and substandard housing arrangements where adhering to social distancing rules was not possible. The COVID-19 pandemic has given new impetus to the debates on socio-spatial inequality along ethnic lines, particularly with a spatial focus on London boroughs. The COVID-19 mortality data showed that the Olympic host boroughs, such as Newham, Tower Hamlets, Hackney and Newham, which contains significant number of ethnic minority residents were the worst affected ones in East London. The disproportionate impact of COVID-19 may resonate with a legacy of *de facto* inequality in East London since minoritised communities are more likely to live in overcrowded and substandard households in deprived areas and working in jobs with higher exposure to infectious diseases (4).

According to LLDC's Local Plan (2015–2031), the London legacy 2012 regeneration process will continue until 2031 with a view to minimising the deprivation gap in comparison to the west of London. Drawing on the empirical evidence and the gap between “the intentions” and “what is happening on the ground”, the paper argues that an inclusive bottom-up approach is needed to deliver the remainder of the legacy projects. To contribute to expert-led planning and delivery of the legacy projects, there is a need for advocacy to mobilise ethnic minority communities to enhance their awareness of opportunities to participate in decision making processes at the neighbourhood level. There is some awareness at the community level, such as activities of the artists' community in Hackney Wick, which has a more unified and strong voice to challenge some LLDC undemocratic decisions (e.g., street protests, campaigns via online platforms). So, some collaboration between the ethnic minority residents and the artists' community could mobilise people to rally around to challenge the top-down planning system. There are local NGOs, such as Hub67 in HW and Bromley-by-Bow Centre in BbB, who could advocate for minoritised communities in seeking alternative resident-led socio-spatial transformative changes. Overall, there is a need for regular dialogues among minoritised communities, local councillors and the planning authority to ensure effective and trustworthy decision-making processes at local levels.

With the Olympic-led spatial transformations, the minoritised ethnic communities in the Olympic host boroughs are going through a transition, so the planning authorities need to revisit the collective desires and aspirations of different ethnic groups in order to share local resources equitably. Most importantly, the allocation of new housing provisions should prioritise pre-existing ethnic minority residents instead of allowing excessive new and affluent residents. Currently, the delivery process of “transforming the heart of East London” promise is highly encouraging newcomers with higher socio-economic status, where it was presumed that pre-existing council tenants of some demolished buildings may not return to the new buildings due to a lack of affordable housing (30). Literature review and the empirical findings indicate that the new build housing estates have a few council tenants (30, 41), but more housing needs to be allocated to the socio-economically disadvantaged and minoritised ethnic residents who have been living in crowded and unhealthy accommodations. In addition, more focus should be given to young peoples' engagement in social and economic activities in order to increase their self-esteem. To deliver the legacy-led benefits to the historically disadvantaged minoritised communities, there is a need for more collaboration between local residents and practitioners through residents' active participation and appropriation in upcoming development projects, otherwise the racialised inequalities would continue to disproportionately impact and exclude vulnerable and minoritised communities as it did throughout the COVID-19 pandemic.

## Data Availability

The raw data supporting the conclusions of this article will be made available by the authors, without undue reservation.

## Footnotes

^1^ https://www.ethnicity-facts-figures.service.gov.uk/

^2^ https://www.newscientist.com/article/2307207-covid-19-death-figures-reveal-huge-ongoing-impact-on-minority-groups/

^3^ https://www.ons.gov.uk/peoplepopulationandcommunity/birthsdeaths andmarriages/deaths/articles/updatingethniccontrastsindeathsinvolving thecoronaviruscovid19englandandwales/10january2022to16february2022

^4^ https://www.ons.gov.uk/peoplepopulationandcommunity/birthsdeaths andmarriages/deaths/bulletins/deathsinvolvingcovid19bylocalareasand deprivation/deathsoccurringbetween1marchand17april

^5^https://news.sky.com/video/coronavirus-virus-does-not-discriminate-gove-11964771#
